# Feasibility of a mini-pig model of radiation-induced brain injury to one cerebral hemisphere

**DOI:** 10.1186/s13014-021-01753-1

**Published:** 2021-02-06

**Authors:** Ilektra Athanasiadi, Whitney D. Perez, Jeannie M. Plantenga, Yava Jones-Hall, Carlos J. Perez-Torres

**Affiliations:** 1 Department of Small Animal Clinical Sciences, Virginia-Maryland College of Veterinary Medicine, Blacksburg, VA USA; 2 School of Health Sciences, Purdue University, 550 Stadium Mall Drive, Hampton Hall 1263A, West Lafayette, IN 47907-2051 USA; 3 Department of Veterinary Clinical Sciences, Purdue University, West Lafayette, IN USA; 4 Purdue University Center for Cancer Research, Purdue University, West Lafayette, IN USA; 5 Department of Comparative Pathobiology, Purdue University, West Lafayette, IN USA

**Keywords:** Radiation-induced brain injury, Radiation leukoencephalopathy, Animal models, Mini-pig

## Abstract

**Background:**

Radiation-induced brain injury is a common concern for survivors of adult and pediatric brain cancer. Pre-clinically, rodent models are the standard approach to evaluate mechanisms of injury and test new therapeutics for this condition. However, these rodent models fail to recapitulate the radiological and histological characteristics of the clinical disease.

**Methods:**

Here we describe a hemispheric mini-pig model of radiation-induced brain injury generated with a clinical 6 MV photon irradiator and evaluated with a clinical 3T MRI. Two pairs of Yucatan mini-pigs each received either 15 Gy or 25 Gy to the left brain hemisphere. Quality of intensity modulated radiation therapy treatment plans was evaluated retrospectively with parameters reported according to ICRU guidelines. The pigs were observed weekly to check for any outright signs of neurological impairment. The pigs underwent anatomical MRI examination before irradiation and up to 6 months post-irradiation. Immediately after the last imaging time point, the pigs were euthanized and their brains were collected for histopathological assessment.

**Results:**

Analysis of the dose volume histograms showed that 93% of the prescribed dose was delivered to at least 93% of the target volume in the left hemisphere. Organs at risk excluded from the target volume received doses below clinical safety thresholds. For the pigs that received a 25 Gy dose, progressive neurological impairment was observed starting at 2 months post-irradiation leading to the need for euthanasia by 3–4 months. On MRI, these two animals presented with diffuse white matter pathology consistent with the human disease that progressed to outright radiation necrosis and severe brain swelling. Histology was consistent with the final MRI evaluation. The pigs that received a 15 Gy dose appeared normal all the way to 6 months post-irradiation with no obvious neurological impairment or lesions on MRI or histopathology.

**Conclusion:**

Based on our results, a mini-pig model of radiation-induced brain injury is feasible though some optimization is still needed. The mini-pig model produced lesions on MRI that are consistent with the human disease and which are not seen in rodent models. Our data shows that the ideal radiation dose for this model likely lies between 15 and 25 Gy.

## Background

Radiation therapy is an integral component in the treatment of intracranial tumors [1, 2]. The use of advanced technologies has allowed for the delivery of higher doses of radiation to areas of the brain that are not accessible to surgery while sparing more normal tissues. Although radiation therapy has helped to improve brain cancer prognosis [1, 2], the side effects caused by radiation are recognized to be significantly associated with decreased quality of life in brain cancer survivors, especially survivors of childhood brain cancer [3]. Late-onset radiation effects, which occur months to years after therapy and do not self-resolve, are the primary concern in terms of radiation-induced brain toxicity. Late-onset radiation-induced brain injury can be categorized into two broad types based on their radiological characteristics: focal and diffuse lesions [4]. Radiation necrosis is usually a focal injury that presents as a mass lesion with focal neurologic abnormalities and evidence of elevated intracranial pressure, whereas cognitive impairment is characterized by diffuse white matter injury [4, 5]. Radiation necrosis and diffuse white matter injury have specific and distinct histological and MRI characteristics [6].

Prior work completed by our lab on the mouse model of radiation-induced diffuse white matter injury showed weaknesses in reproducing the exact brain injury seen in humans [7]. Although histology of the mouse brains revealed a dose-dependent change in the white matter tracts, the changes observed were subtle. Furthermore, we were unable to detect any abnormalities in T1-weighted and T2-weighted MRI images for any dose at any time point after irradiation [7]. These shortcomings, as well as the structural differences between mouse (lissencephalic) and human (gyrencephalic) brains, encouraged our investigation of other animal models with brain tissue characteristics closer to those of humans. Additionally, larger animal species are advantageous when investigating intracranial MRI diagnostic approaches since these are now tested on clinical devices with the same protocols that can directly be used on human patients. The anatomy and size of porcine brains are well suited to address these challenges. Pigs can therefore be used to more accurately model the development of radiation-induced brain injury (RIBI).

Though there are prior reports of a pig model of RIBI [8, 9], these prior works have limited clinical relevance due to how radiation was delivered (electrons instead of photons) and how the model was assessed (non-standard MRI approach). Therefore, the objective of the current study is to establish the feasibility of a RIBI pig model with equipment and approaches that reflect the current clinical scenario. Here, we describe the methods to generate a single-hemisphere RIBI model that is assessed in vivo with a standard clinical MRI approach. The advantage of this model is that all procedures are performed with clinical devices and following the same quality assurance that is performed on patients. Unlike rodent models, this pig model shows changes in anatomical MRI consistent with human RIBI. The approach described can be further adapted to either a whole-brain irradiation model with or without fractionation, or specific focal models targeting or avoiding substructures of interest, e.g., the hippocampus.

## Methods

### Animals and weekly observation:

All animal procedures were approved by the Purdue Animal Care and Use Committee under protocol number 1712001655. Four male 3-month old Yucatan mini-pigs were obtained from Premier BioSource (formerly S&S Farms). All pigs were housed in pairs in a facility designated for large animal research. Water was provided ad libitum and a commercial feed ration was made available twice daily. Pigs were observed at least weekly for any overt neurological impairment. Within the first week after irradiation they were observed every 24 h to ensure no acute side effects. Weights were tacked weekly by animal care staff and showed normal weight gain. Pigs were assessed in two sets of two with the first receiving 25 Gy and the second set receiving 15 Gy. There was no explicit control group, instead the contralateral hemsphere was intended to serve as an internal control for each animal. Animal procedures were performed with the help of the Purdue Pre-Clinical Research Laboratory, a core facility of the Purdue Center for Comparative Translational Research with ample experience with pig models.

### Anesthesia protocol

All pigs were anesthetized using a combination of tiletamine-zolazepam (3 mg/kg), detomidine (0.18 mg/kg), and butorphanol (0.12 mg/kg) administered intramuscularly. After attaining lateral recumbency, pigs were intubated with an appropriately sized endotracheal tube as determined using body weight. General anesthesia was maintained with isoflurane (1–2% inhaled) and oxygen as delivered using mechanical ventilation. Vital signs were monitored and logged throughout all procedures. All pigs received intravenous fluids (PlasmaLyte®; 5–10 mL/kg) via an intravenous catheter in the auricular vein. Butorphanol (0.2 mg/kg IV) was dosed as needed. Pigs were monitored after each procedure to ensure proper recovery from anesthesia until they were capable of standing and walking on their own.

### Immobilization devices and CT simulation

Each anesthetized pig was positioned in sternal recumbency and immobilized using an individualized bite plate [10] and thermoplastic mask on an indexable frame (Uniframe Baseplate, Civco Medical Solutions, Orange City, IA) for radiation therapy simulation CT. The CT simulation treatment couch was positioned in the gantry and the reference isocenter was determined using CT lasers. Crosshair marks were applied to the mask using cloth tape and permanent marker over the intersection of the CT laser at three points. Radiopaque fiducial markers were affixed to the mask at the 3 laser intersection points (Suremark, Vision Line Premium Labels, V-25, Van Arsdale, Innovative Products, Pensacola, FL). Scans were acquired without contrast using a 64-slice CT scanner and 0.625 mm slice thickness (VCT 64-Slice, GE Healthcare, Milwaukee, WI).

### MRI procedure

Subsequent to the CT scan, the pigs were imaged using a 3 T MRI unit (MAGNETOM® Prisma, Siemens Medical Solutions, Malvern, PA) using a 64-channel head coil with the pigs in sternal recumbency under general anesthesia. MR images of the brain were acquired 1 week pre-irradiation, 3 months post-irradiation, and either 4 months (P2) or 6 months (P3 and P4) post-irradiation with a consistent protocol. Included in the protocol were T1-weighted and T2-weighted images acquired using a three-dimensional Magnetization Prepared Rapid Acquisition Gradient Recalled Echo (3D MP-RAGE; TE = 4.7 ms, TR = 2080 ms, averages = 1) sequence and three-dimensional Fast Spin Echo (3D FSE; TE = 410 ms, TR = 2800 ms, averages = 1) sequence, respectively. All scans were acquired with 0.7 mm isotropic resolution with the same geometry. The animals were then given an intravenous injection of 0.2 mL/kg of MultiHance. A period of 11 min was allotted to allow the contrast enough time to accumulate within the intracranial space before acquiring the post-contrast T1-weighted images.

### Radiation treatment planning

CT and MRI images were imported and co-registered using the Varian Eclipse treatment planning system (Varian Eclipse v. 11.0, Varian Medical Systems, Palo Alto, CA). Transverse MRI images and CT images were used for manual brain tissue contouring. The contoured structures included brain, right and left cerebral hemispheres, cerebellum, left and right cerebellum, brainstem, cervical spinal cord, optic nerves (right and left), optic chiasm, eyes, and lenses. Diencephalon was contoured as part of the hemispheres. The planning target volume (PTV) for the first pair of pigs (P1 and P2) included the left cerebral hemisphere and left cerebellum. The PTV for the second pair of pigs (P3 and P4) included the left cerebral hemisphere only. In addition, the structures “brain minus PTV” (brain-PTV) and “brain minus PTV minus 2 mm” were created for plan evaluation and optimization, respectively.

Inverse planning for intensity modulated radiation therapy (IMRT) was used in all pigs. All treatment plans were corrected for tissue heterogeneity using a calculation algorithm (Anisotropic Analytical Algorithm, version 11.0.31, Varian Medical Systems, Palo Alto, CA). For steep dose gradient, the normal tissue objectives were applied using a distance from the target border of 0.1 cm, start dose 100%, end dose 60%, and fall off 0.9 cm. Coplanar, isocentric, non-parallel opposed beams were used with a sliding window technique. Nine angles of radiation beams were distributed entering the left hemisphere (350°, 346°, 330°, 307°, 282°, 270°, 230°, 198°, and 180°). A single dose of 25 Gy for the first pair of pigs (P1 and P2) and 15 Gy for the second pair (P3 and P4) was prescribed to the PTV, while the right side was spared as a control. The single dose of 25 Gy was selected based upon the previous pig model reports [8, 9]. The dose of 15 Gy was chosen based on matching the biological effective dose of one of the most common fractionated whole brain radiotherapy prescriptions (2 Gy × 30 fractions) under the assumption that the alpha–beta ratio of the brain is 3. The plans were evaluated for pre-treatment quality assurance using the MapCheck 2 diode array (Sun Nuclear Corporation, Melbourne, FL). Gamma analysis and distance to agreement analysis were used to compare the planned and output absolute dose with point passing criteria of 3 mm and 3%. The plan was considered acceptable for therapy when at least 95% of all points matched. The evaluation of the plan quality included dose volume histograms (DVHs) and dose color wash for PTV coverage and doses to organs at risk (OARs). The doses to OARs were evaluated according to QUANTEC [11]. RadCalc software (LifeLine Software Inc.) was used as an independent method for verification of the monitor units (MUs). The plans were approved by a veterinary Radiation Oncologist.

The treatment parameters are reported as recommended by the ICRU [12–14]. Briefly, reported treatment parameters for the PTV included maximum (D_2%_), minimum (D_98%_), mean (D_mean_), and median (D_50%_) dose. Homogeneity Index (HI = (D_2%_–D_98%_)/D_50%_), Conformity Index (CI, described below) and Gradient Index (GI = brain volume receiving 50% of prescription dose divided by brain volume receiving 100% of prescription) were used to assess plans retrospectively and were not used in the process of plan approval. An HI close to 0 (zero) shows a homogeneous absorbed dose in the PTV. The CI defines how adequately a target is covered by treatment without irradiation of any tissue outside the PTV. Specifically we calculated the Paddick CI [15] defined as CI = PTV_PIV_^2^/ (PTV × PIV), where PTV_PIV_ is the volume of the PTV that is covered by 100% of the prescription dose and PIV is the brain volume receiving 100% of the prescription dose. A perfect plan has a CI score of 1. The GI is an objective tool to assess how rapidly the dose falls off outside of the PTV. A lower GI indicates steeper dose gradient and a value of < 3 could be ideal. Reported treatment parameters for the OARs (brainstem, cerebellum, spinal cord, optic nerves (right and left), and optic chiasm) included maximum (D_2%_), mean (D_mean_), median (D_50%_), and Volume of Accepted Tolerance Dose (V_ATD_ = dose/volume limit). The maximum point dose (D_max_) was recorded for the lenses. Treatment parameters for the cerebellum were reported only for P3 and P4, since the left side of the cerebellum was included in the PTV for P1 and P2.

### Radiation delivery

Each pig was positioned with the same individualized device used in the CT simulation and aligned to the marked reference isocenter in the radiation therapy vault using room lasers and mask crosshair marks prior to irradiation. Cardinal direction shifts generated in the treatment planning software were applied to align the pig to the plan isocenter. Orthogonal portal MV radiographs were taken to verify the position. A computed portal radiography system was used to develop each portal image (KODAK ACR—2000i, Onconcepts, Rochester, NY). DICOM portal images were imported into the treatment planning system, scaled, and aligned to the digital graticule in the treatment plan’s digitally reconstructed radiographs. The registered images were compared using the offline review program (Varian Medical Systems, Palo Alto, CA). Images were compared for perfect visual alignment of bony structures to the digitally reconstructed radiographs created from CT images used for the IMRT planning. Position was adjusted if alignment differed by greater than 1 mm, and portal radiographs were repeated to document final positioning.

Radiation was delivered with a 6 MV linear accelerator (Varian 6EX, Varian Medical Systems, Inc. Palo Alto, CA) with a 120-leaf multileaf collimator (Millennium 120 MLC, Varian Medical Systems, Palo Alto, CA) using photons with a dose rate of 400 MU/min.

### Necropsy

After the final MRI, pigs were euthanized by intravenous injection with pentobarbital (100–200 mg/kg). Due to neurological deficits, P1 was euthanized at 4 months and P2 at 3 months post irradiation. P3 and P4 were euthanized at 6 months as we had originally planned for all pigs. Brains were extracted by veterinary staff of the Indiana Animal Disease Diagnostic Laboratory and left in 10% neutral buffered formalin for at least 24 h. Coronal gross sections were generated to match areas of interest on the MRI datasets, embedded in paraffin, and stained with hematoxylin and eosin (H&E) and Luxol Fast Blue (LFB). The former was utilized for general pathological examination of the sections while the latter was used to evaluate white matter integrity of the irradiated hemisphere.

## Results

### Quality of half-brain treatment plan

**Fig. 1:**
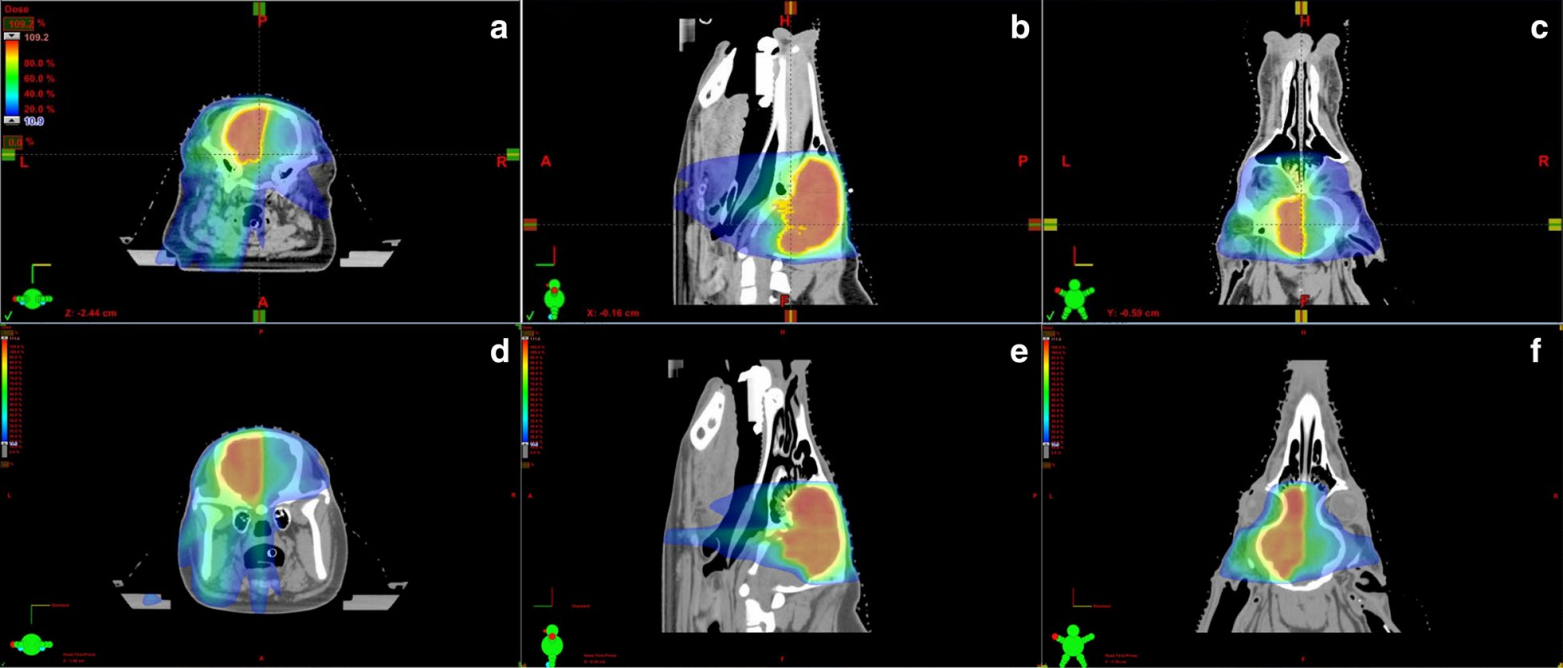
Treatment plan from subjects P1 and P3. Panels **a**–**c** for P1 and Panels **d**–**f** for P3 show a colorwash of the dose being delivered on the transverse (**a** and **d**), sagittal (**b** and **e**), and coronal (**c** and **f**) planes respectively. Blue areas receive ~ 10–20%, green areas ~ 50–60% and red areas ~ 100% of the target dose. The difference in coverage for the cerebellum on these plans can be best appreciated in the sagittal views

The radiation treatment plan for each pig passed the quality assurance as described in the methods. Briefly, for the PTV dose coverage, 93% of the prescribed dose covered at least 93% of the PTV. The dose color wash and DVHs were similar in all 4 pigs. Figure 1 shows an example of dose color wash in 3 planes from P1 and P3. The prescribed dose is homogeneously distributed over the PTV and there is a steep fall-off of the dose at the PTV margins. The dosimetric parameters for the PTV for all pigs are summarized in Table 1. The minimum, maximum, mean, and median doses to the PTV are reported as analyzed by the DVHs. The HI, CI, and GI for all four plans ranged 0.15–0.21, 0.57–0.74, and 1.9–2.7, respectively. The dosimetric parameters for the OARs for all pigs are summarized in Table 2. Briefly, doses to the spinal cord and lenses are much lower than the cut off recommended by QUANTEC for myelopathy or cataract, respectively [11]. Regarding the brainstem the high maximum (D_2%_) doses especially for P1 and P2 were seen as expected at the side adjacent to the PTV. However, the mean doses to the brainstem were low (3–11.3 Gy) in all for pigs. The optic apparatus (right and left optic nerve, optic chiasm) received doses relatively close to the prescribed doses as expected. The left optic nerve and the optic chiasm were included in the PTV and the right optic nerve was adjacent to the PTV. The dosimetric parameters for the cerebellum were reported for P3 and P4. The high maximum (D_2%_) doses were seen as expected adjacent to the PTV and the mean doses (3.4 Gy) were low. Looking more globally at the untreated parts of the brain in the brain-PTV volume, again we see the highest doses adjacent to the PTV but the mean doses are low (30 to 45% of the prescribed dose).

**Table 1:** Summary of dosimetric results for PTV analyzed from dose-volume histogram. D_X%_ = dose (Gy) received by the x% of the volume; D_mean_ = mean dose received by the volume; HI = homogeneity index; CI = conformity index; GI = gradient index

Pigs	Volume (cm^3^)	Min (D_98%_)	Max (D_2%_)	Mean (D_mean_)	Median (D_50%_)	HI	CI	GI
*Summary of dosimetric results for PTV analyzed from dose-volume histogram*								
P1	35.4	22.8	26.8	25.3	25.5	0.16	0.74	1.9
P2	32.3	23	26.9	25.3	25.4	0.15	0.65	2.2
P3	31.0	13.4	16.4	15.1	15.1	0.20	0.57	2.6
P4	26.6	13.0	16.1	15.0	15.1	0.21	0.59	2.7

**Table 2:** Summary of dosimetric results for OARs analyzed from dose-volume histogram

	P1	P2	P3	P4
**Summary of dosimetric results for OARs analyzed from dose-volume histogram**				
*Brainstem (V*_*mean*_ = *3.9 cm*^*3*^*)*				
D_2%_ (Gy)	22.4	17.7	10.1	10.4
D_mean_ (Gy)	11.3	9.6	3.6	3.0
V_10/12_ (%)	53/34	37/20	< 3.4	< 3.3
*Cerebellum (V*_*mean*_ = *4.3 cm*^*3*^*)*				
D_2%_ (Gy)			11.1	10.5
D_mean_ (Gy)			3.4	3.4
V_10/12_ (%)			4.6	4.1
*Optic chiasm (V*_*mean*_ = *0.1 cm*^*3*^*)*				
D_2%_ (Gy)	24.7	23.7	11.0	9.3
D_mean_ (Gy)	22.9	22.0	10.0	6.7
V_6/8/10_ (%)	1.0	1.0	≤ 1.0	< 0.5
*Left optic nerve (V*_*mean*_ = *0.1 cm*^*3*^*)*				
D_2%_ (Gy)	22.1	23.2	10.4	7.4
D_mean_ (Gy)	17.9	17.4	9.2	6.0
V_6/8/10_ (%)	1.0	1.0	≤ 1.0	≤ 0.5
*Right optic nerve (V*_*mean*_ = *0.1 cm*^*3*^*)*				
D_2%_ (Gy)	20.2	19.1	9.0	4.5
D_mean_ (Gy)	16.9	14.2	8.3	3.8
V_6/8/10_ (%)	0.1	0.1	0.1	0.1
*Spinal cord (V*_*mean*_ = *3.7 cm*^*3*^*)*				
D_2%_ (Gy)	5.5	3.3	< 1	< 1
D_mean_ (Gy)	1.0	0.6	0.2	0.2
V_8/10/12_ (%)	< 0.3	0	0	0
*Left lens (V*_*mean*_ = *0.2 cm*^*3*^*)*				
D_max_ (Gy)	3.4	1.4	< 0.1	< 0.1
*Right lens (V*_*mean*_ = *0.2 cm*^*3*^*)*				
D_max_ (Gy)	5.6	2.7	1.7	1.5
*Brain – PTV (P1&P2) (V*_*mean*_ = *30 cm*^*3*^*)*				
D_2%_ (Gy)	22.5	18		
D_mean_ (Gy)	11	7.7		
V_10/12_ (%)	49/35	52/32		
*Brain – PTV (P3&P4) (V*_*mean*_ = *36.6 cm*^*3*^*)*				
D_2%_ (Gy)			10.7	11.4
D_mean_ (Gy)			5.3	5.3
V_10/12_ (%)			≤ 4	≤ 8

D_x%_ = dose received by the x% of the volume; D_mean_ = mean dose received by the volume; V_x_ (V_ATD_) = volume receiving at least x Gy; Vmean = mean structure volume of the four pigs; D_max_ = maximum point dose

### MRI features of the irradiated pig brain

**Fig. 2:**
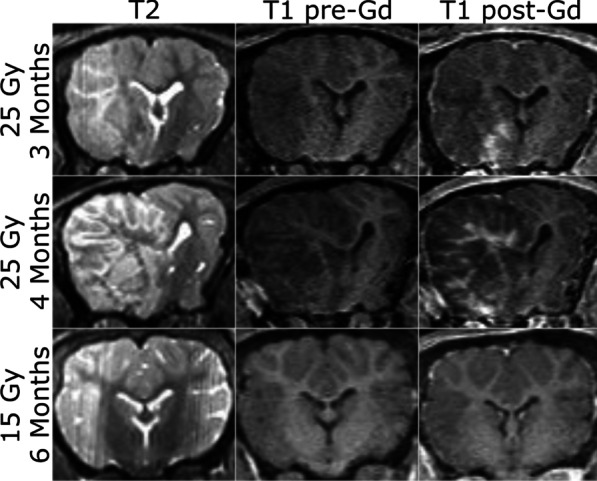
Anatomical MRI after irradiation of the left hemisphere of the mini-pig brain for Pig 1. Diffuse enhancement is seen on T2-weighted imaging with mass effect with foci of enhancement on post-contrast T1-weighted imaging

The original plan was to acquire MR images of the brain at 3 and 6 months post-irradiation on a 3 T scanner to potentially detect early-delayed pathology and late-onset RIBI. Our first group of pigs (P1 and P2) developed obvious neurological deficits (left head tilt and left circling) as early as 2 months post-irradiation. One pig (P2) had to be euthanized at 3 months post-irradiation due to inability to stand, which we believe was due to a lesion observed in the brainstem on MRI (figure not shown). The other pig (P1) was euthanized at 4 months as the neurological deficits grew significantly worse and the pig’s balance was significantly impaired. Anatomical MR images of this second pig show that at 3 months post-irradiation, there is diffuse enhancement on T2-weighted imaging with minimal enhancement on post-contrast T1-weighted imaging (Fig. 2 top row) which is consistent with RIBI but occurs much sooner than expected. By 4 months post-irradiation, the observed pathology became much more extensive based on both T2-weighted and post-contrast T1-weighted enhancement (Fig. 2 middle row), with massive amounts of edema that led to a midline shift and collapse of the lateral ventricle. Our second group of pigs (P3 and P4) showed no signs of neurological deficits throughout the entire 6 month follow-up duration. Anatomical MR images showed no abnormal T2-weighted or post-contrast T1-weighted enhancements at 3 and 6 months post-irradiation for both P3 (Fig. 2 bottom row) and P4.

### Histological features of the irradiated pig brain

**Fig. 3:**
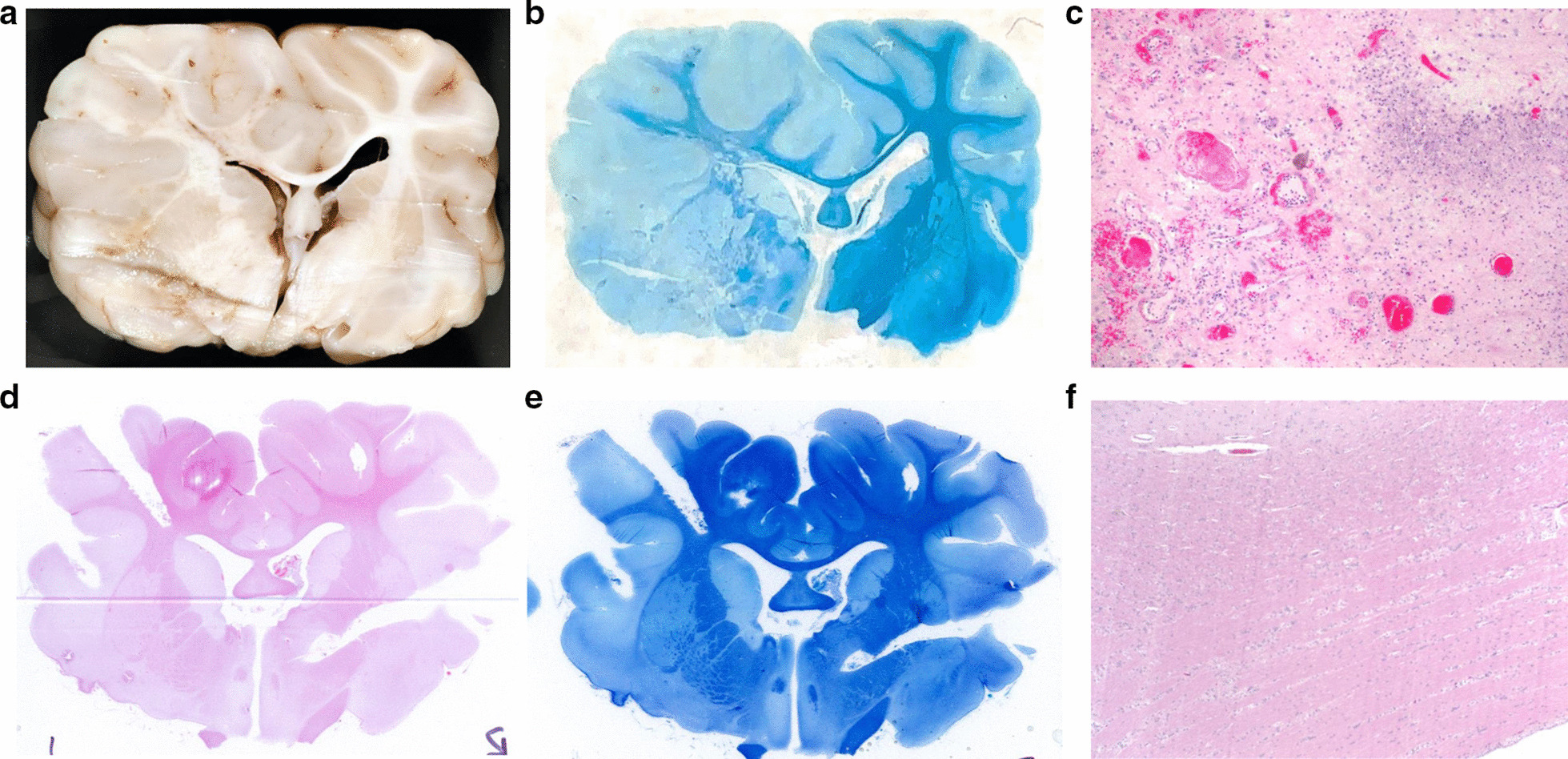
Histological changes in the pig brain after 25 Gy irradiation. The section was chosen to be in roughly the same location as the MRI data shown in Fig. 2. **a** Macroscopic examination is consistent with the MRI findings with mass effect and disrupted white matter. **b** Wide field picture of the Luxol-Fast Blue stained section shows clear demyelination of the left hemisphere as expected of RIBI. **c** × 10 magnification of the hematoxylin and eosin shows vascular changes and inflammatory infiltration suggestive of radiation necrosis. In the case of 15 Gy, wide field pictures of the H&E (**d**) and LFB (E) as well as × 10 magnification of the H&E (**F**) look normal

After euthanasia and macroscopic evaluation we used hematoxylin and eosin (H&E) and Luxol Fast Blue (LFB) staining to validate our MRI findings. Macroscopic examination of the tissue before sectioning confirmed the MRI findings with obvious mass effect shifting the midline, disruption of the white matter, and likely focal hemorrhages. Examination of the H&E sections from the pigs that received a dose of 25 Gy (Fig. 3 top row) evidenced extensive cerebral (mostly unilateral) necrosis with associated inflammation (glial cells and glitter cells), vasculitis, vascular wall necrosis, thrombosis, dystrophic mineralization and loss of myelin. The contralateral hemisphere only had pathology near the midline.Though the lesions are more severe in the white matter, pathology can also be found in the grey matter. The lesions are also not homogenous suggesting that there might be a mixture of both types of pathologies consistent with the MRI. These changes are consistent with has been previously observed histologically in RIBI [16] and radiation necrosis [17]. In contrast, for pigs that received a dose of 15 Gy (Fig. 3 bottom row) no apparent pathological changes were evident on either macroscopic or microscopic examination.

## Discussion

With advancements in radiation therapy techniques and improved efficacy in treating disease, the prognosis and median survival time with inoperable brain tumors is constantly improving [1, 2]. However, this also means that late adverse effects from neurocranial radiation therapy are becoming increasingly recognized [18]. Establishing an improved animal model for RIBI is imperative to facilitate the development of treatments for RIBI in both human and veterinary medicine. The use of clinical standard radiotherapy and MR imaging protocols in our study not only allows for the treatment and diagnosis of RIBI in our pig model, but also increases the translational value of the model. The model presented here improves upon the common rodent models in that pathology can be detected in MRI like in human patients. Our model also improves on prior work in pigs as that work relied on an electron beam for irradiation which led to an overestimation of the dose delivered to the brain.

Here, we present a pig model of RIBI generated with an IMRT half-brain treatment plan. The radiation treatment provides a homogeneous dose distribution throughout the PTV leading to pathology limited to the PTV as evidenced on clinically standard MRI and histopathology. As expected, late-onset RIBI pathology was detected primarily in the irradiated hemisphere but only for those animals that received 25 Gy. RIBI pathology on MRI became more severe over time, which is consistent with previous descriptions of RIBI [18]. These results demonstrate that our clinical methodology is well-suited to produce late-onset RIBI pathology that is restricted to the irradiated regions of the pig brain. Prior reports in pig models [8, 9], were limited by the use of a 12 meV electron beam without correction for the skull which led to an overestimation of dose delivered to the brain. Our approach uses clinically relevant 6 MV photons with standard treatment planning technique (IMRT), clinical validation, and quality assurance to ensure the dose delivered is the dose that was planned. In comparison to established rodent models [7, 19], in this pig model we can detect abnormalities with anatomical MRI consistent with standard clinical approaches. Furthermore, white matter damage detected by H&E and LFB staining is not only more obvious than previously observed in the mouse brain, but it is also correlated with the anatomical lesions detected by MRI. Together, these data suggest that IMRT is a feasible treatment delivery for a pre-clinical pig model of late-onset RIBI that is more accurate than current rodent models.

## Conclusion

While this work shows that a mini-pig model of RIBI is feasible, there remains some details that need to be improved upon. Work still needs to be done to optimize the target radiation dose and the time that observations are made to comprehensively study the development of diffuse white matter lesions, which have been observed in RIBI [20, 21]. Although a 25 Gy dose was able to produce clear MRI pathology in both P1 and P2, the onset was earlier than expected and the pathology progressed to radiation necrosis with unacceptably severe neurological impairment. However, a 15 Gy dose was unable to induce any MRI abnormalities or histopathology up to 6 months post-irradiation. This suggests that a suitable dose to produce diffuse lesions within a pig brain lies within the window of 15 Gy to 25 Gy. Additionally, it will be important to assess cognitive deficits in this model and how it relates to the lesions detected on MRI and histology. An additional constraint of our current results is the use of a hemispheric model where the unirradiated hemisphere serves as an internal control. A whole-brain model may be more ideal for evaluation of cognitive deficits. A major advantage of the larger pig brain and our approach is the possibility to generate very specific irradiation plans of brain substructures. This could be leveraged for a more precise model of hippocampal-avoidance whole brain radiotherapy.

## Data Availability

The datasets used and/or analyzed during the current study are available from the corresponding author on reasonable request.

## Abbreviations

CI: conformity index
CT: computed tomography
DVH: dose volume histogram
GI: gradient index
H&E: hematoxylin and eosin
HI: homogeneity index
ICRU: International Commission on Radiation Units and Measurements
LFB: luxol fast blue
IMRT: intensity modulated radiation therapy
MRI: magnetic resonance imaging
OAR: organ at risk
PTV: planning target volume
RIBI: radiation-induced brain injury
